# The Railways of the World

**Published:** 1883-01

**Authors:** 


### THE RAILWAYS OF THE WORLD.

The lines of railways in the five divisions of the earth cost, in round numbers, $16,000,000,000 ; and would, according to Baron Kolb, reach eight times round the globe, although it is but little over half a century since the first railway worked by steam was opened between Darlington and Stockton, Sept. 27, 1825, and between Manchester and Liverpool, Sept. 15, 1830. It is shown that in France, previous to the existence of railways, there was one passenger in every 335,000 killed, and one out of every 30,000 wounded ; whereas between 1835 and 1875, there was but one in 5,178,890 killed, and one in 580,450 wounded ; so that we may infer that the tendency to accidents is yearly diminishing. Railway traveling in England is attended with greater risk than in any other country in Europe. A French statistician observes that, if a person were to live continually in a railway carriage, and spend all his time in railway traveling, the chances of his dying from a railway accident would not occur till he was 960 years old.

